# Influence of Positive End-Expiratory Pressure Titration on the Effects of Pronation in Acute Respiratory Distress Syndrome: A Comprehensive Experimental Study

**DOI:** 10.3389/fphys.2020.00179

**Published:** 2020-03-12

**Authors:** Gaetano Scaramuzzo, Lorenzo Ball, Fabio Pino, Lucia Ricci, Anders Larsson, Claude Guérin, Paolo Pelosi, Gaetano Perchiazzi

**Affiliations:** ^1^Department of Morphology, Surgery and Experimental Medicine, University of Ferrara, Ferrara, Italy; ^2^Hedenstierna Laboratory, Department of Surgical Sciences, Uppsala University, Uppsala, Sweden; ^3^Department of Surgical Sciences and Integrated Diagnostics, University of Genoa, Genoa, Italy; ^4^San Martino Policlinico Hospital, IRCCS for Oncology and Neurosciences, Genoa, Italy; ^5^Groupement Hospitalier Centre, Médecine Intensive Réanimation, Hospices Civils de Lyon, Lyon, France; ^6^Université de Lyon, Université Claude Bernard Lyon 1, Villeurbanne, France; ^7^INSERM 955 – Eq13, Institut Mondor de Recherche Biomédicale, Créteil, France; ^8^Department of Anesthesia, Operation and Intensive Care Medicine, Akademiska Sjukhuset, Uppsala, Sweden

**Keywords:** acute respiratory distress syndrome, positive end-expiratory pressure, prone positioning, titration, ventilator-induced lung injury

## Abstract

Prone position can reduce mortality in acute respiratory distress syndrome (ARDS), but several studies found variable effects on oxygenation and lung mechanics. It is unclear whether different positive end-expiratory pressure (PEEP) titration techniques modify the effect of prone position. We tested, in an animal model of ARDS, if the PEEP titration method may influence the effect of prone position on oxygenation and lung protection. In a crossover study in 10 piglets with a two-hit injury ARDS model, we set the “best PEEP” according to the ARDS Network low-PEEP table (BP_ARDS_) or targeting the lowest transpulmonary driving pressure (BP_DPL_). We measured gas exchange, lung mechanics, aeration, ventilation, and perfusion with computed tomography (CT) and electrical impedance tomography in each position with both PEEP titration techniques. The primary endpoint was the PaO_2_/FiO_2_ ratio. Secondary outcomes were lung mechanics, regional distribution of ventilation, regional distribution of perfusion, and homogeneity of strain derived by CT scan. The PaO_2_/FiO_2_ ratio increased in prone position when PEEP was set with BP_ARDS_ [difference 54 (19–106) mmHg, *p* = 0.04] but not with BP_DPL_ [difference 17 (−24 to 68) mmHg, *p* = 0.99]. The transpulmonary driving pressure significantly decreased during prone position with both BP_ARDS_ [difference −0.9 (−1.5 to −0.9) cmH_2_O, *p* = 0.009] and BP_DPL_ [difference −0.55 (−1.6 to −0.4) cmH_2_O, *p* = 0.04]. Pronation homogenized lung regional strain and ventilation and redistributed the ventilation/perfusion ratio along the sternal-to-vertebral gradient. The PEEP titration technique influences the oxygenation response to prone position. However, the lung-protective effects of prone position could be independent of the PEEP titration strategy.

## Introduction

Prone positioning is an established therapeutic option for patients affected by severe acute respiratory distress syndrome (ARDS) (ARDS Definition Task Force et al., 2012) for its effects on gas exchange, lung protection (Douglas et al., 1977; Mure et al., 1997), and mortality (Mancebo et al., 2006; Guérin et al., 2013). Oxygenation is improved during prone positioning by ventilation increase in the vertebral lung, resulting from a more homogeneous distribution of the ventilation/perfusion ratio across lung regions (Richter et al., 2005). On the other hand, the beneficial effects on ventilator-induced lung injury (VILI) are explained by vertebral lung recruitment, reduced hyperinflation of the ventral regions, and homogenization of regional ventilation (Perchiazzi et al., 2011).

Despite several clinical studies reporting oxygenation improvement, the benefits on mortality were conflicting (Gattinoni et al., 2001; Guérin et al., 2004; Taccone et al., 2009): mortality was reduced mainly in severe ARDS (Sud et al., 2010), and pronation-induced oxygenation improvement was not associated with improved survival (Albert et al., 2014). This could be due to the distinct mechanisms mediating the effects of pronation on gas exchange and lung protection. Moreover, the method to titrate positive end-expiratory pressure (PEEP) was not consistent across the trials; therefore, it is unknown whether the method used to set PEEP affects the clinical benefits of PP.

In this crossover study in a porcine model of ARDS, we investigated the physiologic effects of prone positioning while setting PEEP with two different methods: the low PEEP/FiO_2_ ARDS Network table and the lowest transpulmonary driving pressure. We hypothesized that the PEEP titration method influences the effects of pronation on oxygenation, lung aeration, ventilation, and perfusion.

## Materials and Methods

The study was approved by the Regional Animal Ethics Committee (Uppsala, Sweden, protocol C24/16; decision date 2016-03-18), and the care and handling of the animals were in accord with the National Institutes of Health guidelines for ethical animal treatment. Following the principles of reduction and refinement in animal experimentation (Russell and Burch, 1959), the data retrieved from the animals involved in this experiment were also analyzed for the purposes of other studies, not interfering with the present one and not yet published. The experiments were planned according to the PREPARE guidelines (Smith et al., 2018) and were performed at the Hedenstierna Laboratory, Uppsala University, Sweden.

### Instrumentation and Monitoring

Ten pigs [32 (31–33) kg] were positioned supine and premedicated with Zoletil Forte (tiletamine and zolazepam) 6 mg kg^−1^ and xylazine 2.2 mg kg^−1^ i.m. and anesthetized with i.v. infusion of ketamine 30 mg kg^−1^ h^−1^, midazolam 0.1 mg kg^−1^ h^−1^, and fentanyl 3.75 μg kg^−1^ h^−1^. After assessment of the adequacy of anesthesia—defined as unresponsiveness to painful stimulation between the front hooves—muscle relaxation was obtained with a continuous i.v. infusion of rocuronium 0.3 mg kg^−1^ h^−1^. After a bolus of fentanyl 10–20 μg kg^−1^, an 8 mm inner diameter endotracheal tube (Mallinckrodt Medical, Ireland) was positioned through a surgical anterior neck access. An arterial line was placed in the right carotid artery, and a central venous line was placed in the right jugular internal vein through surgical access. A pulmonary artery catheter (Criti Cath, Ohmeda Pte Ltd., Singapore) was introduced via an external jugular vein, and its correct position was verified by pressure monitoring. Cardiac output (CO) was measured in triplicate with the thermodilution technique (Siemens SC 9000XL, Dräger, Germany). During the experiment, airway pressure (Paw) and flow (f) were continuously recorded at the airway opening. Esophageal pressure (Pes) was measured with an esophageal balloon (CooperSurgical, Inc., Trumbull, USA) inserted through the oral route; its correct position was verified according to Baydur et al. (1982). Paw and Pes were measured by pressure transducers (Digima Clic pressure transducers; Special Instruments, Nördlingen, Germany). Flow was recorded by a Fleisch pneumotachograph (Laminar Flow Element type PT; Special Instruments, Germany) positioned between the endotracheal tube and the ventilator and connected to a differential pressure transducer (Diff-Cap pressure transducer; Special Instruments, Nördlingen, Germany). Signals from the transducers were forwarded to an analog-to-digital converter card (DAQ-card AI-16XE50; National Instruments, Austin, TX, USA) with a sampling frequency of 200 Hz, using the BioBench Software (National Instruments, Austin, TX, USA). A 32-electrode electrical impedance tomography (EIT) belt was placed around the shaved chest of the animal and connected to a dedicated monitor (Enlight 1800, Timpel, Brazil). The signal quality was checked at the beginning and constantly monitored during the experimental protocol. During instrumentation, the animals were ventilated using volume-controlled ventilation (VCV) mode with a Servo-I ventilator (Getinge, Solna, Sweden) with tidal volume (VT) 6 ml kg^−1^, PEEP 5 cmH_2_O, inspiratory to expiratory ratio (I:E) 1:2, respiratory rate (RR) 30 bpm, and FiO_2_ 0.4.

### Lung Injury Model

After instrumentation, a two-hit lung injury was used to create an ARDS model: the first hit was warm saline lung lavage (20 mkg^−1^), repeated until PaO_2_/FiO_2_ (P/F) ratio was <150 mmHg. Thereafter, 120 min of injurious mechanical ventilation (PEEP 2 cmH_2_O, inspiratory pressure 35 cmH_2_O, I:E 1:2, and RR 20 bpm) was started (Borges et al., 2015). Once the injurious model was established, the animals were stabilized for 30 min in VCV (VT 6 ml kg^−1^, PEEP 5 cmH_2_O, and RR 20 bpm).

### Investigation Protocol

The protocol was divided into the following three consecutive steps: (1) PEEP titration, (2) gas exchange and respiratory mechanics, and (3) lung imaging. During the whole protocol, 5 min was allowed to elapse before measurements after each change of position.

#### Positive End-Expiratory Pressure Titration

Two different PEEP titration trials were performed sequentially, one using the ARDSnet low PEEP/FiO_2_ table and one based on the lowest transpulmonary driving pressure. Specifically, the ARDSnet table uses a combination of PEEP and FiO_2_ to reach a target SpO_2_ (88–95%) (Brower et al., 2004); the titration was made in the supine position, increasing progressively PEEP/FiO_2_ until the SpO_2_ target was reached. The PEEP level found during this trial was defined as BP_ARDS_. Afterward, a decremental PEEP titration trial was conducted starting from PEEP 20 cmH_2_O to PEEP 0 cmH_2_O in steps of 1 cmH_2_O. At each PEEP step, end-inspiratory and end-expiratory pauses were performed to obtain the values of airway pressure and esophageal pressure at end-inspiration (EIaw and EIeso) and end-expiration (EEaw and EEeso), from which the corresponding transpulmonary pressures were computed (EEL and EIL), as follows:

$$\begin{array}{l} \text{EEL}=\text{EEaw}-\text{EEeso}\\ \text{ EIL}=\text{EIaw}-\text{Eieso} \end{array}$$

The lung driving pressure (ΔPL) was calculated as EIL – EEL. The level of PEEP corresponding to the lowest ΔPL was defined as best PEEP (BP_DPL_); the PEEP titration trial was conducted in both prone and supine positions, thus defining a BP_DPL_ in supine (BP_DPL_,S) and prone (BP_DPL_,P) position. Before each titration trial, the positioning was followed by a recruitment maneuver (RM) (pressure-controlled ventilation, peak inspiratory pressure 40 cmH_2_O, PEEP 5 cmH_2_O; I:E 1:1; and RR 6 bpm for 1 min) to standardize the lung history. During both trials, VT was set to 6 ml kg^−1^ and RR = 20 bpm.

#### Gas Exchange and Respiratory Mechanics

The following four steps were randomly applied with a Latin square crossover design: (1) supine, BP_ARDS_; (2) supine, BP_DPL,S_; (3) prone, BP_ARDS_; and (4) prone, BP_DPL,P_. An RM was performed before each step, and during the different steps, RR and VT were kept constant at 30 bpm and 6 ml kg^−1^, respectively. In the prone position, BP_ARDS_ step, the PEEP level found in the titration trial in supine position was used. FiO_2_ was kept constant in both BP_ARDS_ and BP_DPL_ between supine and prone positions. At the end of each period, the following were measured: (1) arterial blood gas analysis (ABL 500 Radiometer, Copenhagen, Denmark); (2) static respiratory mechanics; (3) EIT signal of 20 representative breaths before and after CVC injection of 10 ml of hypertonic saline (NaCl 10%), to obtain ventilation and perfusion distribution images (Borges et al., 2012). Mechanical power (MP) was assessed using the following equation (Gattinoni et al., 2016):

$$\begin{array}{l} Power_{rs}\\ =0.098\;\cdot \;RR\;\cdot \{VT^{2}\cdot \left[\frac{1}{2}\cdot EL_{rs}+RR\;\cdot \frac{\left(1\;+\;I\;:\;E\right)}{60\;\cdot \;I\;:\;E}\cdot \;R_{aw}\right]\;\\ +\;VT\;\cdot \;PEEP\} \end{array}$$

#### Image Acquisition and Analysis

Animals were transported to the CT facility where the ventilation sequence was repeated for the CT image acquisition. Images were acquired during inspiratory and expiratory holds with a Somatom Force scanner (Siemens, Germany) and reconstructed in 5 mm slices with a B41 convolution kernel. Images were manually segmented with ITKSnap (http://www.itksnap.org) to select the lung parenchyma excluding big vessels and airways. The analysis was carried out using purposely written MatLab scripts (Image Processing Toolbox, The MathWorks, Natick, MA, USA; Version R2018) on the whole lung and on a regional basis, dividing the parenchyma into four ventro-dorsal regions of interest with equal lung mass content (ROI1 = sternal region to ROI4 = vertebral region), as previously described (Güldner et al., 2016). Regional strain was calculated in each ROI dividing the CT-assessed variation of volume by the regional end-expiratory lung volume. To evaluate the regional strain heterogeneity, a coefficient of variation was calculated for each step of the trial, as follows:

$$\begin{array}{l} CV_{strain\;}=\;\frac{{SD\;strain}_{\left(ROI1,\;ROI2,\;ROI3,\;ROI4\right)}}{{Mean\;strain}_{\left(ROI1,ROI2,\;ROI3,\;ROI4\right)}} \end{array}$$

We computed tidal recruitment as the difference between non-aerated lung tissue (%) from expiration to inspiration, as previously described (Güldner et al., 2016).

### Electrical Impedance Tomography Imaging

The EIT images were analyzed trough a purposely made MatLab script. To allow inter-animal comparisons, in each EIT image, the variation of impedance related to ventilation (ΔZ_V_) was normalized to the highest pixel level ΔZ_V_, yielding values ranging from 0 to 1. To assess lung perfusion, a bolus of hypertonic saline (NaCl 10%, 10 ml) was injected intravenously using the distal tip of the central vein catheter during an expiratory hold maneuver. The variation of impedance related to the saline passage through the pulmonary circulation was recorded and graphically reconstructed in a 32 × 32 pixel EIT image, as previously described (Borges et al., 2012). The relative pixel variation of impedance was normalized to the maximum pixels' value to allow inter-animal comparisons. The global inhomogeneity index of ventilation (GIv) was calculated using the following formula (Zhao et al., 2014):

$$\begin{array}{l} GIv=\;\frac{\sum_{x,y\in lung\;}|{\Delta Zv}_{xy}-Median\left({\Delta Zv}_{lung\;}\right)|}{\sum_{nx,y\in lung}{\Delta Zv}_{xyn}} \end{array}$$

To evaluate heterogeneity in perfusion assessed by EIT, impedance maps related to perfusion were analyzed using the same process. The variation of impedance related to perfusion (ΔZp) was normalized to the highest pixel level ΔZp, yielding values ranging from 0 to 1. The global inhomogeneity index of perfusion (GIp) was therefore calculated as follows:

$$\begin{array}{l} GIp=\;\frac{\sum_{x,y\in lung\;}|{\Delta Zp}_{xy}-Median\left({\Delta Zp}_{lung\;}\right)|}{\sum_{nx,y\in lung}{\Delta Zp}_{xyn}} \end{array}$$

The EIT derived regional compliance was calculated both for the respiratory system and for the lungs, assuming that ΔZv = ΔV, as previously described (Spadaro et al., 2018). Region of interest division was made using the mass content derived from the corresponding CT scans. For regional analysis of EIT ventilation and perfusion data, we assumed that the vertebral–dorsal gradient observed in the EIT slice was representative of the whole lung. For the analysis of ventilation at the ROI level, we computed for each ROI the estimated specific ventilation, measured in milliliters of tidal volume per minute per gram of lung tissue, using the following formula:

$$\begin{array}{l} Ventilation_{ROI}=\frac{\frac{{\Delta Z}_{v,\;ROI}}{{\Delta Z}_{v,\;lung}}{\cdot V}_{T}\cdot RR}{{Lung\;mass}_{ROI}} \end{array}$$

where ΔZ_V_ is the variation of impedance related to ventilation, V_T_ the tidal volume, and RR the respiratory rate. Similarly, we computed for each ROI the estimated specific perfusion, measured in milliliters of blood flow per minute per gram of lung tissue, using the following formula:

$$\begin{array}{l} Perfusion_{ROI}=\frac{\frac{{\Delta Z}_{p,\;ROI}}{{\Delta Z}_{p,\;lung}}\cdot CO}{{Lung\;mass}_{ROI}} \end{array}$$

where ΔZ_p_ is the variation of impedance related to perfusion and CO is the cardiac output in milliliters per minute. Finally, we computed the ventilation perfusion ratio for each ROI as follows:

$$\begin{array}{l} \frac{\overset{∙}{V}}{Q}=\frac{Ventilation_{ROI}}{Perfusion_{ROI}} \end{array}$$

### Statistical Analysis

The sample size calculation was based on a previously published study (Retamal et al., 2016), from which we expected a P/F ratio of 115 ± 5.5 mmHg in a double-hit model of ARDS pigs. In this crossover study design, we expected a strong within-subject correlation (*R* > 0.6) between the P/F ratio in prone and supine positions, and we considered clinically relevant an increase of 10%. This increase corresponds to an effect size of 1.41; therefore, accounting for the asymptotic relative efficiency of non-parametric tests, we needed to include at least eight animals to achieve 90% power (1 – β) to detect such difference at an α level of 0.05. To compensate for a potential drop-off rate of 20%, we aimed to include 10 pigs. Values are expressed as median (first to third quartiles). We compared continuous variables using the Friedman test (non-parametric analysis of variance for repeated measurements) with the Dunn *post-hoc* test (prone vs. supine position using the same PEEP titration technique, two comparisons). For continuous variables with missing measurements, to maximize the use of data, we used a mixed-effects model comprising positioning as a dichotomous fixed effect with a fixed interaction (positioning within PEEP titration mode) and animal as a random effect with a random intercept. The correlation between variables was assessed using the Spearman correlation for non-parametric data. The statistical analysis was carried out using SPSS 19 and GraphPad Prism 6. Statistical significance was set at *p* < 0.05.

### Electrical Impedance Tomography Missing Files

Owing to unavailability of EIT machine or major artifacts in perfusion assessment, we had 30% (12/40) missing data points for ventilation maps and 53% (21/40) for perfusion maps.

## Results

The baseline characteristics of the animals are summarized in Supplement Table 1. In the supine position, PaO_2_/FiO_2_ after the two-hit injury and the RM was 150 (123–155) mmHg, and the respiratory system driving pressure was 13 (13–14) cmH_2_O. BP_ARDS_ was 10.0 (8.0–10.0) cmH_2_O in both positions, whereas BP_DPL,S_ was 12.5 (11.3–13.0) cmH_2_O and BP_DPL,P_ was 10.5 (10.0–11.0) cmH_2_O. There was no intra-subject correlation between BP_DPL,S_ and BP_DPL,P_ (ρ = 0.21; *p* = 0.56).

### Gas Exchange, Hemodynamics, and Respiratory Mechanics

**Figure 1:**
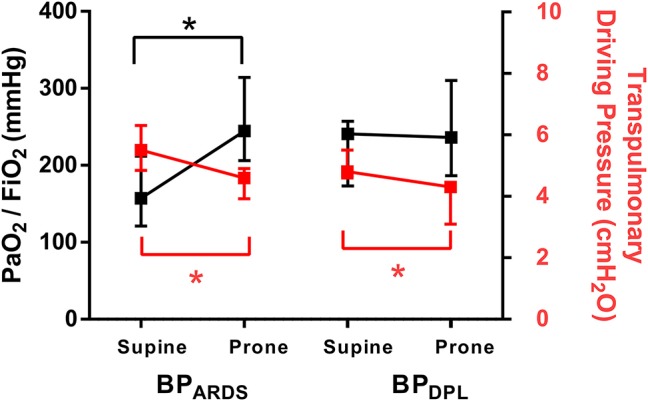
Effect of prone positioning on PaO_2_/FiO_2_ ratio (black, left *y*-axis) and on transpulmonary driving pressure (red, right *y*-axis) in both PEEP titration techniques. Symbols are median (25–75th percentile). *p < 0.05. PEEP: positive end-expiratory pressure; BP_ARDS_ PEEP titrated according to the ARDS Network low PEEP/FiO_2_ table; BP_DPL_ PEEP set according to the lowest transpulmonary driving pressure.

An overview of gas exchange, hemodynamics, and respiratory mechanics in the different positioning/PEEP combinations is provided in Table 1. PaO_2_/FiO_2_ increased in prone vs. supine position with BP_ARDS_ [difference 54 (19–106) mmHg, *p* = 0.04], but not with BP_DPL_ [difference 17 (−24 to 68) mmHg, *p* = 0.99]. The respiratory system driving pressure decreased during prone positioning with both BP_ARDS_ [difference −1.0 (−2.0 to 0.0) cmH_2_O, *p* = 0.03] and BP_DPL_ [difference −1.8 (−2.0 to 1.00) cmH_2_O, *p* = 0.03]. The transpulmonary driving pressure significantly decreased during prone position with both BP_ARDS_ [difference −0.9 (−1.5 to −0.9) cmH_2_O, *p* = 0.009] and BP_DPL_ [difference −0.55 (−1.6 to −0.4) cmH_2_O, *p* = 0.04] (Figure 1). Change in PaO_2_/FiO_2_ did not correlate with change in respiratory system driving pressure from supine to prone position, neither with BP_ARDS_ (ρ = 0.28; *p* = 0.47) nor with BP_DPL_ (ρ = 0.08; *p* = 0.83). The MP was not reduced with prone positioning (*p* = 0.60 and *p* = 0.11 with BP_ARDS_ and BP_DPL_, respectively).

**Table 1:** Gas exchange, hemodynamics, and lung mechanics parameters in prone and supine positions with the two PEEP titration strategies in 10 pigs.

	**PEEP set according to the** **ARDS Network low PEEP/FiO**_****2****_ **table (BP**_**ARDS**_**)**		**PEEP set targeting the** **lowest transpulmonary driving pressure (BP**_**DPL**_**)**		***p*****-value**		
	**Supine**	**Prone**	**Supine**	**Prone**	**ANOVA^†^**	**BP_**ARDS**_^‡^**	**BP_**DPL**_^‡^**
**Gas exchange**							
PaO_2_ (mmHg)	90 (79–100)	171 (107–211)	122 (103–174)	143 (97–205)	0.02	0.04^*^	0.99
PaO_2_/FiO_2_ (mmHg)	157 (127–199)	245 (214–302)	241 (192–250)	236 (194–293)	0.02	0.04^*^	0.99
PaCO_2_ (mmHg)	70 (65–72)	69 (64–76)	66 (61–70)	70 (62–81)	0.60		
pHa	7.24 (7.18–7.27)	7.25 (7.20–7.26)	7.24 (7.21–7.25)	7.21 (7.19–7.29)	0.64		
**Hemodynamics**							
Cardiac output (L min^−1^)	4.0 (3.2–5.4)	4.2 (3.1–4.6)	4.0 (2.6–4.9)	4.3 (3.2–4.5)	0.14		
Mean arterial pressure (mmHg)	80 (72–97)	79 (64–94)	75 (62–81)	82 (61–90)	0.25		
Systolic pulmonary artery pressure (mmHg)	38 (33–40)	35 (33–38)	37 (32–39)	38 (32–39)	0.69		
**Lung mechanics**							
Tidal volume (mL)	193 (190–200)	193 (190–200)	193 (190–200)	193 (190–200)	>0.99		
Peak airway pressure (cmH_2_O)	21.5 (19.5–23.5)	20.5 (17.5–21.8)	23.0 (20.5–27.0)	20.0 (19.0–22.3)	<0.001	0.04^*^	0.009^*^
Plateau airway pressure (cmH_2_O)	17.5 (16.3–19.5)	17.0 (14.3–17.8)	19.5 (17.0–22.5)	16.5 (15.3–17.8)	0.002	0.24	0.006^*^
Positive end-expiratory pressure (cmH_2_O)	10.0 (8.0–10.0)	10.0 (8.0–10.0)	12.5 (11.3–13.0)	10.5 (10.0–11.0)	0.003	>0.99	0.11
Driving pressure of the respiratory system (cmH_2_O)	8.0 (6.8–9.3)	7.0 (5.6–7.3)	7.0 (6.0–8.3)	6.0 (4.4–7)	<0.001	0.03^*^	0.03^*^
Mechanical power (J min^−1^)	9.9 (8.7–11.9)	9.5 (8.2–11.3)	11.1 (10.0–12.6)	10.2 (9.0–10.7)	0.006	0.60	0.11
End-inspiratory transpulmonary pressure (cmH_2_O)	3.8 (2.8–4.2)	3.5 (3.1–4.4)	5 (4.5–5.5)	4 (3.1–4.5)	0.02	0.99	0.03^*^
End-expiratory transpulmonary pressure (cmH_2_O)	−2.1 (−3.8 to −1.2)	−1.1 (−2.1 to 1.2)	0.2 (−1.0 to 0.8)	−0.5 (−1.1 to 0.3)	0.004	0.08	0.99
Transpulmonary driving pressure (cmH_2_O)	5.5 (4.9–6.3)	4.6 (3.9–4.9)	4.8 (4.6–5.5)	4.3 (3.1–4.5)	<0.001	0.009^*^	0.039^*^
Respiratory system resistance (cmH_2_O L^−1^ s^−1^)	13 (11–15)	13 (9.9–18)	13 (10–18)	14 (11–17)	0.98		
Respiratory system elastance (cmH_2_O L^−1^)	41 (33–49)	36 (27–39)	38 (29–43)	30 (22–37)	<0.001	0.03^*^	0.03^*^
Chest wall elastance (cmH_2_O L^−1^)	11 (5.5–17)	11 (6–14)	11 (3.4–18)	8.8 (6.9–15)	0.95		
Lung elastance (cmH_2_O L^−1^)	28 (25–31)	24 (19–26)	25 (24–28)	22 (16–23)	<0.001	0.009^*^	0.04^*^
Elastance-derived transpulmonary pressure (cmH_2_O)	11.8 (10.4–15.9)	11.4 (10.3–14.7)	12.6 (10.5–20)	11.6 (10.0–16.8)	0.33		

*PEEP, positive end-expiratory pressure; ARDS Network, Acute Respiratory Distress Syndrome Network*.

† *Friedman test*.

‡ *Dunn post hoc comparing prone vs. supine position*.

* *Significant difference between prone and supine using the same PEEP titration technique (p < 0.05)*.

### Global Aeration, Ventilation, and Perfusion Analysis

Lung aeration data derived from CT scan analysis are reported in Table 2. With BP_ARDS_, and hence at same PEEP, prone position increased normally aerated lung tissue (*p* = 0.019) and decreased strain (*p* = 0.019). With BP_DPL_, prone position did not increase normally aerated tissue (*p* > 0.99), increased non-aerated tissue (*p* < 0.001) and did not reduce strain (*p* = 0.77). There was no correlation between the PaO_2_/FiO_2_ variation and the strain variation from supine to prone position with BP_ARDS_ (ρ = 0.30; *p* = 0.43) or BP_DPL_ (ρ = 0.47; *p* = 0.17). Ventilation and perfusion EIT data are reported in Table 3. Prone positioning shifted the center of ventilation toward vertebral direction (*p* = 0.006 and *p* < 0.001 with BP_ARDS_ and BP_DPL_, respectively) and reduced the GIv (*p* = 0.001 and *p* < 0.001 with BP_ARDS_ and BP_DPL_, respectively) but had no effect on the GIp or on the center of perfusion.

**Table 2:** Computed tomography lung aeration data.

	**PEEP set according to the** **ARDS Network low PEEP/FiO**_**2**_ **table (BP**_**ARDS**_**)**		**PEEP set targeting the** **lowest transpulmonary driving pressure (BP**_**DPL**_**)**		***p*****-value**		
	**Supine**	**Prone**	**Supine**	**Prone**	**ANOVA^†^**	**BP_**ARDS**_^‡^**	**BP_**DPL**_^‡^**
Total lung volume (ml)	1,429 (1,287–1,442)	1,462 (1,357–1,531)	1,642 (1,536–1,660)	1,563 (1,443–1,631)	0.003	0.24	0.45
Total lung mass (g)	788 (70–864)	785 (692–885)	801 (720–900)	782 (702–889)	0.013	0.98	0.08
Gas fraction (%)	0.45 (0.42–0.47)	0.48 (0.46–0.49)	0.50 (0.47–0.55)	0.51 (0.45–0.53)	0.001	0.049^*^	0.77
Lung strain	0.35 (0.31–0.42)	0.32 (0.26–0.37)	0.3 (0.24–0.31)	0.28 (0.26–0.34)	0.006	0.019^*^	0.77
Hyperaerated tissue (%)	0.0 (0.0–0.2)	0.0 (0.0–0.2)	0.0 (0.0–0.0)	0.0 (0.0–0.0)	0.13		
Normally aerated tissue (%)	30.1 (25.4–34.9)	40.8 (36.1–44.1)	44.2 (34.5–53.2)	41.7 (36.0–47.9)	0.003	0.019^*^	>0.99
Poorly aerated tissue (%)	63.1 (61.9–64.3)	46.4 (45.1–48.0)	53.9 (44.0–59.5)	47.6 (44.0–50.5)	<0.001	<0.001^*^	0.24
Non-aerated tissue (%)	6.4 (4.0–9.2)	11.9 (9.9–16.9)	3.2 (1.3–5.3)	7.7 (4.9–15.5)	<0.001	0.11	<0.001^*^
Tidal recruitment (%)	0.6 (−0.9 to 4.0)	0.1 (−0.8 to 4.3)	0.9 (0.3–2.1)	2.4 (1.1–3.8)	0.15		

*CT, computed tomography; PEEP, positive end-expiratory pressure; ARDS Network, Acute Respiratory Distress Syndrome Network*.

† *Friedman test*.

‡ *Dunn post hoc comparing prone vs. supine position. Except for tidal recruitment, values correspond to the average between inspiratory and expiratory CT scan*.

* *Significant difference between prone and supine using the same PEEP titration technique (p < 0.05)*.

**Table 3:** Electrical impedance tomography global lung ventilation and perfusion data.

	**PEEP set according to the** **ARDS Network low PEEP/FiO**_**2**_ **table (BP**_**ARDS**_**)**		**PEEP set targeting the** **lowest transpulmonary driving pressure (BP**_**DPL**_**)**		***p*****-value**		
	**Supine**	**Prone**	**Supine**	**Prone**	**Positioning^†^**	**BP_**ARDS**_^‡^**	**BP_**DPL**_^‡^**
Global inhomogeneity of ventilation (GIv)	86.3 (85.5–89.4)	85.5 (84.3–89.5)	87.7 (86.5–89.5)	84.5 (83.5–87.3)	<0.001	0.006^*^	<0.001^*^
Global inhomogeneity of perfusion (GIp)	83.9 (81.2–84.7)	82.4 (78.7–83.2)	84.5 (81.7–86.3)	81.9 (80.0–88.8)	0.61	0.83	0.62
Center of ventilation	44.1 (43.3–46.2)	63.1 (60.3–64.9)	41.4 (37.0–49.7)	59.0 (51.2–64.4)	<0.001	0.001	<0.001^*^
Center of perfusion	55.0 (48.4–56.3)	52.1 (47.9–56.2)	50.3 (43.9–52.6)	50.4 (44.0–51.1)	0.56	0.64	0.72

*EIT, electrical impedance tomography. Mixed-effects model analysis for global EIT data*.

† *Positioning effect*.

‡ *Contrast estimate significance*.

* *Significant difference between prone and supine using the same PEEP titration technique (p < 0.05). Centers of ventilation and perfusion are expressed ranging from 0 to 100, where 0 is most sternal and 100 most vertebral*.

### Regional Aeration, Ventilation, and Perfusion Analysis

**Figure 2:**
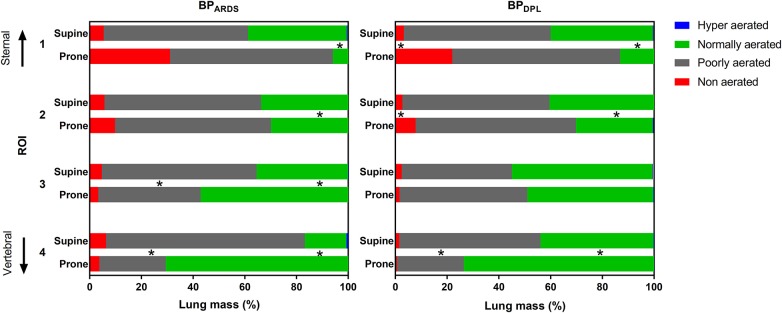
Computed tomography regional aeration analysis. Bars represent the size of the aeration compartments in supine and prone positions (averaged between inspiratory and expiratory CT scan) with PEEP set according to BP_ARDS_ (left panel) and BP_DPL_ (right panel). Values are median (25th−75th percentile). *p < 0.05 for the corresponding aeration compartment between prone and supine using the same PEEP titration technique.

**Figure 3:**
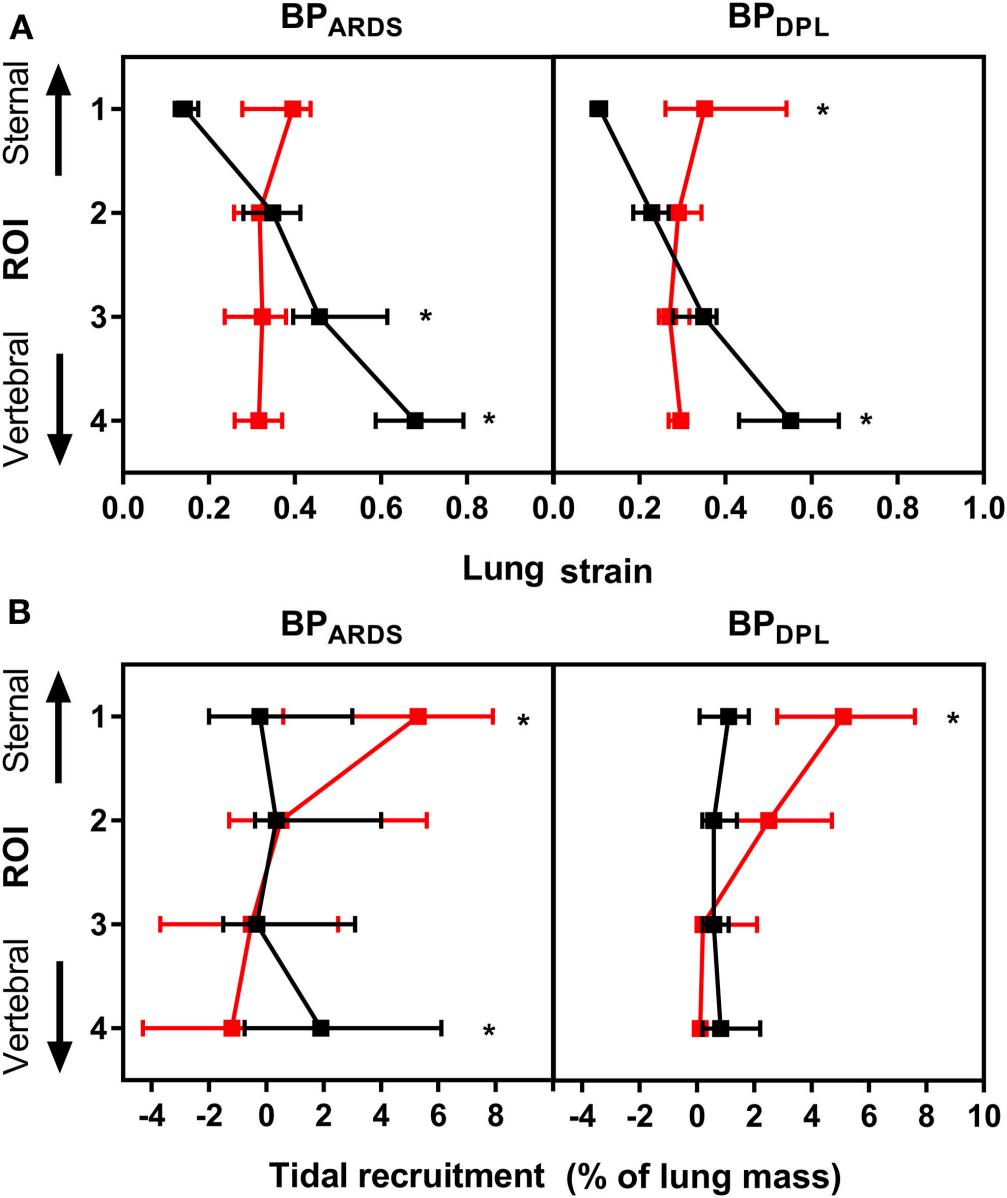
CT-derived regional strain **(A)** and tidal recruitment **(B)** in supine (black dots) and prone (red dots) positions with PEEP set according to BP_ARDS_ (left panels) and BP_DPL_ (right panels). Data plotted as median (25–75th percentile). *Significant difference in the same ROI in prone vs. supine with *p* < 0.05. CT, computed tomography; ROI, region of interest.

**Figure 4:**
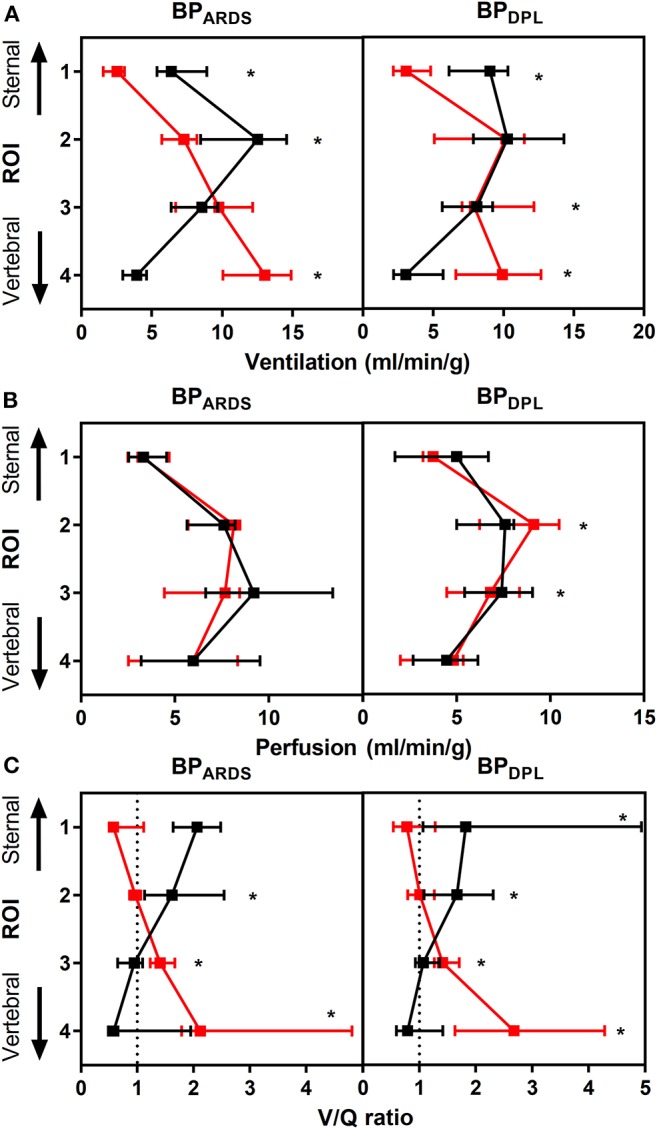
EIT-derived ventilation **(A)**, perfusion **(B)**, and ventilation/perfusion ratio **(C)** data in supine (black dots) and prone (red dots) positions with PEEP set according to BP_ARDS_ (left panels) and BP_DPL_ (right panels). Data plotted as median (25–75th percentile). *Significant difference in the same ROI in prone vs. supine with *p* < 0.05. ROI: region of interest; EIT: electrical impedance tomography; PEEP: positive end-expiratory pressure; BP_ARDS_ PEEP titrated according to the ARDS Network low PEEP/FiO_2_ table; BP_DPL_ PEEP set according to the lowest transpulmonary driving pressure.

Detailed data on regional CT and EIT analyses are reported in Supplement Tables 2, 3. As illustrated in Figure 2, with BP_ARDS_, prone position shifted normally aerated tissue from sternal to vertebral ROIs and decreased poorly aerated tissue in the two most vertebral ROIs (*p* < 0.001). With BP_DPL_, prone position increased normally aerated tissue in the vertebral ROIs (*p* = 0.004), whereas it increased non-aerated tissue in mid-sternal (*p* = 0.006) and sternal (*p* < 0.001) ROIs. The regional compliance of the respiratory system improved in the vertebral ROI in prone vs. supine position with both BP_ARDS_ (*p* = 0.003) and BP_DPL_ (*p* = 0.043), whereas it was unchanged elsewhere. As shown in Figure 3, strain was distributed more homogeneously between ROIs in prone position with both BP_ARDS_ [coefficient of variation from 52 (49–56)% in supine to 21 (12–42)% in prone, *p* < 0.001] and BP_DPL_ [from 55 (52–63)% to 19 (12–35)%, *p* < 0.001]. With BP_ARDS_, in prone positioning, a tidal recruitment increase was observed in the sternal ROI (*p* = 0.03), with a symmetric reduction in the vertebral ROI (*p* = 0.019). With BP_DPL_, prone positioning increased tidal recruitment in the sternal ROI (*p* = 0.011). In prone position, regional ventilation assessed with EIT was redistributed from sternal to vertebral regions in both BP_ARDS_ and BP_DPL_ (Figure 4A), whereas perfusion was marginally affected only with BP_DPL_ (Figure 4B). The ventilation/perfusion ratio increased in vertebral and decreased in sternal regions (Figure 4C). With BP_ARDS_, the ROIs with a ventilation/perfusion ratio below 1 were 2/4 (50%) and 1/4 (25%) in supine and prone positions, respectively. With BP_DPL_, 1/4 (25%) ROIs had a ventilation/perfusion ratio below 1 in both prone and supine positions. Prone position did not reduce the coefficient of variation across ROIs of ventilation, perfusion, and ventilation/perfusion ratio regardless of the PEEP titration technique (see Supplementary Table 4).

## Discussion

In this study, we tested the effects of prone position on gas exchange, lung mechanics, aeration, and perfusion when PEEP was set according to two different titration techniques in a porcine model of early ARDS. We found that prone position (1) improved oxygenation when PEEP was set according to the ARDS Network low PEEP/FiO_2_ titration table but not when PEEP was set according to the lowest transpulmonary driving pressure obtained in a decremental PEEP trial; (2) reduced the respiratory system and lung driving pressure in both PEEP titration techniques; (3) increased aeration of vertebral lung in both titration techniques and homogenized regional lung strain; and (4) redistributed ventilation and the ventilation/perfusion ratio distribution as assessed by EIT.

Our results show that the PEEP titration technique can influence the response in oxygenation after pronation. It is well-known that prone position does not determine a uniform effect on gas exchange in all ARDS patients (Sud et al., 2010; Haddam et al., 2016). In our model, we performed an RM before setting ventilation in each PEEP/position combination. When combined with BP_DPL_, this corresponds to applying a modified “open lung approach” PEEP setting method (Lachmann, 1992), which can improve oxygenation substantially in early ARDS with high recruitment potential through the use of PEEP levels higher than those used when targeting oxygenation alone. This could explain why we did not find further gas exchange improvement with prone position with such PEEP titration technique (Figure 1, right panel). In fact, prone positioning increased lung aeration with BP_ARDS_, but not for with BP_DPL_, where significant increase of non-aerated tissue was observed in the sternal ROI. Of note, we titrated PEEP on the basis of the lowest transpulmonary pressure also in prone position, which is not yet validated. In a recent experimental study, esophageal manometry in supine provided a good estimation of regional transpulmonary pressure acting on dependent lung regions adjacent to the esophageal balloon (Yoshida et al., 2018). In prone position, the sternal dependent lung region is distant from the point where esophageal pressure is measured, possibly leading to underestimation of the PEEP level required to maximize recruitment. It has been reported that pronation improves oxygenation in most but not all patients with ARDS (Mure et al., 1997; Gattinoni et al., 2001; Guérin et al., 2004; Mancebo et al., 2006; Taccone et al., 2009): our results suggest that the method used to titrate PEEP could interact with the effects of pronation, potentially explaining the heterogeneous response observed among patients and in clinical trials.

We also investigated several global and regional parameters previously associated with the development of VILI: respiratory system and transpulmonary driving pressure, MP, elastance-derived transpulmonary pressure (stress), strain, and tidal recruitment. Prone position reduced both respiratory system and transpulmonary driving pressures with each PEEP titration technique. MP is a recently introduced measurement, combining several components of VILI in one metric (Tonetti et al., 2017; Russotto et al., 2018); it was not affected by prone positioning in our study, independent of PEEP titration, as it was the case for lung stress. The global lung strain was reduced by pronation only with BP_ARDS_. Nonetheless, the regional strain was redistributed more homogeneously across ROIs regardless of the PEEP titration method. This reflects a more even distribution of V_T_ on the regional end-expiratory lung volumes, as already reported by Mentzelopoulos et al. (2005) in severe ARDS patients. With BP_ARDS_, cyclic tidal recruitment shifted from vertebral to sternal regions, whereas with BP_DPL_, it increased in the sternal region but did not decrease in the vertebral ROI. These findings suggest that even though pronation did not improve oxygenation in BP_DPL_, prone position could protect the lungs independent of the method used to titrate PEEP, by means of reduction of the driving pressure and homogenization of lung strain. Of note, we did not observe a correlation between gas exchange improvement and driving pressure reduction: this strengthens the concept that the lung-protective effect of prone position is independent of oxygenation improvement (Albert et al., 2014).

EIT ventilation and perfusion analysis figured out a marginal effect of prone positioning on perfusion redistribution between ROIs: the main changes in the ventilation/perfusion ratio were attributable to the redistribution of ventilation. The GIv decreased significantly in both PEEP titration techniques, meaning that intratidal ventilation is more evenly distributed across the lung.

These findings highlight two important issues. First, PEEP and pronation act independently on gas exchange and lung aeration, confirming the recent findings by Keenan et al. (2018). Second, gas exchange and protective mechanical ventilation are unlinked phenomena, explaining why an increase in oxygenation following pronation was not able to predict mortality reduction in patients with ARDS (Albert et al., 2014).

Our paper has several limitations. First, the respiratory mechanics measurements and the CT scans were not done simultaneously, owing to the inability to perform the whole experiment in the CT facility. However, we performed RMs between each ventilation step to restore lung history. Furthermore, the physiologic and lung imaging parts of our study have internal consistency. Second, the experiment was conducted on an animal model of ARDS, and further studies are needed to confirm these data on patients. Third, it is a short-term study, and whether or not present findings are maintained over time should be assessed. Fourth, the presence of missing data limits the possibility of drawing definitive conclusions from the interpretation of EIT results, although the application of mixed-model effects should limit this problem. Therefore, further studies are needed to confirm our findings. Fifth, the levels of PaCO_2_ were consistently high during the experiment, although no differences were found among the different steps. RR and tidal volume, in fact, were unchanged and not adjusted during the trial, to keep constant the weight of these parameters on MP calculation. Finally, the same level of PEEP was used in BP_ARDS_ conditions in both prone and supine positions because no evidence exists about the need of re-adjustment after prone positioning when the PEEP/FiO_2_ table is used to titrate PEEP.

## Conclusions

The oxygenation response to prone positioning depends on the PEEP titration technique, and it may not be seen when using driving transpulmonary pressure to set PEEP. Furthermore, prone positioning could enhance lung protection independently from PEEP titration technique and from the oxygenation response.

## Data Availability Statement

The datasets generated for this study are available on request to the corresponding author.

## Ethics Statement

The animal study was reviewed and approved by Regional Animal Ethics Committee (Uppsala, Sweden, protocol C24/16; decision date 2016-03-18).

## Author Contributions

GS, LB, and FP conducted the study, analyzed the data, and wrote the manuscript. LR conducted the image segmentation and the preliminary analysis. AL, PP, CG, and GP designed the study, supervised the protocol, interpreted the data, and revised the manuscript.

### Conflict of Interest

The authors declare that the research was conducted in the absence of any commercial or financial relationships that could be construed as a potential conflict of interest.
