# Supplementary material for: Influence of Positive End-Expiratory Pressure Titration on the Effects of Pronation in Acute Respiratory Distress Syndrome: A Comprehensive Experimental Study
Source: Front Physiol. 2020 Mar 12;11:179. doi: 10.3389/fphys.2020.00179 (PMC7080860; doi:10.3389/fphys.2020.00179)
Supplement: Supplementary file 1 [file Table_1.DOCX]

**Influence of PEEP titration on the effects of pronation in ARDS: a comprehensive experimental study - Online supplement**

Gaetano Scaramuzzo, Lorenzo Ball, Fabio Pino, Lucia Ricci, Anders Larsson, Claude Guerin, Paolo Pelosi, Gaetano Perchiazzi

**Supplement Table 1: Baseline characteristics**

| Weight (kg) | 32 [31 – 33] |
| --- | --- |
| V_T_ (ml) | 193 [190 - 200] |
| PEEP (cmH_2_O) | 10 [8 - 10] |
| FiO_2_ | 0.5 [0.5 - 0.7] |
| Ppeak (cmH_2_O) | 28 [26 - 29] |
| Pplat (cmH_2_O) | 24 [22 - 24] |
| Minute ventilation (L/min) | 5.9 [5.7 - 6.0] |
| Respiratory system driving pressure (cmH_2_O) | 13 [13 - 14] |
| SpO_2_ (%) | 92 [91 - 92] |
| EtCO_2_ (%) | 57 [46 - 71] |
| PaO_2_ (mmHg) | 81 [78 - 85] |
| PaCO_2_ (mmHg) | 70 [60 - 83] |
| PaO_2_/FiO_2_ (mmHg) | 150 [123 - 155] |
| Mean arterial pressure (mmHg) | 71 [66 - 82] |
| Heart rate (beats/min) | 99 [89 - 108] |
| Cardiac output (l/min) | 4.0 [3.5 - 5.0] |

Baseline characteristics after lung injury. Values are median [1st-3^rd^ quartiles]. Definition of abbreviations: V_T_ = tidal volume, PEEP = positive end expiratory pressure, FiO_2_ = fraction of oxygen in air, SpO_2_ = transcutaneous oxygen saturation, EtCO_2_ = end tidal CO_2_, Ppeak = peak airway pressure, Pplat = plateau pressure, PaO_2_ = partial pressure of oxygen in arterial blood, PaCO_2_ = partial pressure of carbon dioxide in arterial blood.

**Supplement Table 2. Regional lung aeration**

|  | ***ROI*** | ***PEEP set according to the***  ***ARDS Network low PEEP/FiO_2_ table (BP_ARDS_)*** | | ***PEEP set targeting the***  ***lowest transpulmonary driving pressure (BP_DPL_)*** | | ***P value*** | | |
| --- | --- | --- | --- | --- | --- | --- | --- | --- |
|  |  | ***Supine*** | ***Prone*** | ***Supine*** | ***Prone*** | ***ANOVA***^†^ | ***BP_ARDS_***^‡^ | ***BP_DPL_***^‡^ |
| ROI lung volume (ml) | 1 | 381 [339-440] | 273.5 [227-296] | 409 [368-464] | 265 [254-308] | <0.001 | 0.004* | <0.001* |
|  | 2 | 320 [295-359] | 293 [280-315] | 369 [325-397] | 307 [293-338] | <0.001 | 0.011* | 0.004* |
|  | 3 | 324 [313-356] | 368 [363-394] | 391 [362.2-414] | 381 [370-437] | 0.09 | 0.019* | >0.99 |
|  | 4 | 300 [279-331] | 434 [402-515] | 368 [330-407] | 480 [434-539] | <0.001 | <0.001* | 0.002 |
| ROI lung mass (g) | 1 | 186 [169-206] | 191 [168-212] | 190 [172-214] | 189 [171-217] | 0.11 |  |  |
|  | 2 | 186 [166-202] | 184 [162-209] | 189 [169-211] | 184 [164-208] | 0.034 | 0.97 | 0.08 |
|  | 3 | 185 [166-204] | 182 [163-205] | 190 [169-210] | 178 [166-206] | 0.029 | 0.77 | 0.019* |
|  | 4 | 191 [171-209] | 185 [163-209] | 192.2 [174-219] | 185 [167-209] | 0.003 | 0.075 | 0.011* |
| Gas fraction (%) | 1 | 0.53 [0.47-0.54] | 0.27 [0.23-0.33] | 0.54 [0.51-0.56] | 0.32 [0.28-0.37] | <0.001 | 0.006* | <0.001* |
|  | 2 | 0.46 [0.39-0.50] | 0.42 [0.37-0.44] | 0.49 [0.45-0.55] | 0.44 [0.36-0.47] | <0.001 | 0.019* | 0.002* |
|  | 3 | 0.45 [0.41-0.50] | 0.55 [0.49-0.56] | 0.53 [0.50-0.55] | 0.52 [0.49-0.58] | 0.02 | 0.004* | 0.60* |
|  | 4 | 0.34 [0.32-0.40] | 0.60 [0.57-0.61] | 0.48 [0.41-0.52] | 0.61 [0.58-0.63] | <0.001 | <0.001* | 0.004* |
| Hyper aerated tissue  (% of ROI lung mass) | 1 | 0.27 [0.26-0.29] | 0.31 [0.30-0.36] | 0.20 [0.20-0.20] | 0.26 [0.24-0.30] | 0.003 | 0.24 | 0.019* |
|  | 2 | 0.29 [0.27-0.32] | 0.30 [0.29-0.33] | 0.25 [0.20-0.26] | 0.27 [0.26-0.29] | <0.001 | >0.99 | 0.17 |
|  | 3 | 0.31 [0.27-0.32] | 0.26 [0.24-0.29] | 0.23 [0.21-025] | 0.24 [0,14-0,31] | 0.017 | 0.98 | 0.60 |
|  | 4 | 0.29 [0.27-0.31] | 0.19 [0.14-0.23] | 0.26 [0.25-0.28] | 0.15 [0.09-0.23] | 0.002 | 0.011* | 0.031* |
| Normally aerated tissue  (% of ROI lung mass) | 1 | 38.2 [32.4-39.1] | 7.6 [6.2-13.6] | 39.5 [37.6-44.2] | 11.3 [11-18.5] | <0.001 | 0.006* | <0.001* |
|  | 2 | 35.2 [21-43.8] | 26.9 [15.7-31.5] | 40.6 [31.2-53.9] | 27.7 [16.5-36] | <0.001 | 0.049* | 0.006* |
|  | 3 | 35.4 [19.6-45.7] | 58.4 [44-66.6] | 54.3 [44.3-62.9] | 46.7 [43.86-67.85] | 0.002 | 0.004* | 0.60 |
|  | 4 | 12.1 [8.3-21.1] | 72.2 [66.8-74] | 43.7 [24.2-56.1] | 73.6 [69.8-77.9] | <0.001 | <0.001* | 0.004* |
| Poorly aerated tissue  (% of ROI lung mass) | 1 | 54.8 [52.7-56] | 62.99 [49.8-67] | 55.7 [51.7-57.9] | 62.9 [48.5-67.9] | 0.81 |  |  |
|  | 2 | 60.6 [51.2-68.2] | 60.3 [57-62.2] | 56.9 [43.6-62.9] | 60 [58.2-65.3] | 0.24 |  |  |
|  | 3 | 59.8 [48.4-68.7] | 39.6 [30.1-48.1] | 42.6 [35.1-51.7] | 48.4 [30.8-52.8] | 0.002 | <0.001* | 0.98 |
|  | 4 | 76.2 [70.3-84.0] | 25.7 [21.2-28.1] | 54.6 [43.3-66.8] | 25.8 [21.6-28.7] | <0.001 | <0.001* | 0.011* |
| Non aerated tissue  (% of ROI lung mass) | 1 | 5.4 [3.6-10.8] | 31.1 [22.4-33.9] | 3.3 [1.6-4.3] | 22 [13.1-37.1] | <0.001 | 0.11 | <0.001* |
|  | 2 | 5.7 [3.5-8.0] | 9.8 [7-15.1] | 2.7 [1.8-6.4] | 7.8 [2.8-18.3] | 0.002 | 0.11 | 0.006* |
|  | 3 | 4.7 [2.6-6.6] | 3.3 [2.9-5.4] | 2.4 [1.7-3.8] | 1.6 [1.2-4.8] | 0.086 |  |  |
|  | 4 | 6.31 [4.29-11.49] | 3.72 [3.10-4.71] | 1.49 [0.43-2.48] | 0.59 [0.38-1.02] | <0.001 | 0.77 | 0.45 |
| Tidal recruitment  (% of ROI lung mass) | 1 | -0.2 [-2.0 - 3.0] | 5.3 [0.6 - 7.9] | 1.1 [0.1 - 1.8] | 5.1 [2.8 - 7.6] | 0.002 | 0.03* | 0.011* |
|  | 2 | 0.3 [-0.4 - 4.0] | 0.5 [-1.3 - 5.6] | 0.6 [0.2 - 1.4] | 2.5 [0.5 - 4.7] | 0.57 |  |  |
|  | 3 | -0.3 [-1.5 - 3.1] | -0.5 [-3.7 - 2.5] | 0.6 [0.2 - 1.1] | 0.2 [0.0 - 2.1] | 0.13 |  |  |
|  | 4 | 1.9 [-0.8 - 6.1] | -1.2 [-4.3 - 1.9] | 0.8 [0.2 - 2.2] | 0.1 [0.1 - 0.7] | 0.02 | 0.019* | 0.45 |

^†^ Friedman test; ^‡^ Dunn post-hoc comparing prone vs. supine position. Except tidal recruitment, values correspond to the average between inspiratory and expiratory CT scan. *Significant difference between prone and supine using the same PEEP titration technique (p < 0.05). CT: computed tomography; ROI: region of interest (1-vertebral … 4-dorsal); PEEP: positive end-expiratory pressure; ARDS Network: Acute Respiratory Distress Syndrome Network.

**Supplement Table 3. Regional lung EIT analysis**

|  | ***ROI*** | ***PEEP set according to the***  ***ARDS Network low PEEP/FiO_2_ table (BP_ARDS_)*** | | ***PEEP set targeting the***  ***lowest transpulmonary driving pressure (BP_DPL_)*** | | ***P value*** | | |
| --- | --- | --- | --- | --- | --- | --- | --- | --- |
|  |  | ***Supine*** | ***Prone*** | ***Supine*** | ***Prone*** | **Position**^†^ | ***BP_ARDS_***^‡^ | ***BP_DPL_***^‡^ |
| Regional compliance of the  respiratory system (ml/cmH_2_O) | 1 | 5.2 [4.6-7.1] | 4 [2.7-6.4] | 7.5 [5.4-12] | 4.1 [2.9-8.4] | 0.25 | 0.74 | 0.21 |
|  | 2 | 7.3 [5.8-14] | 7.3 [ 6.7-8] | 7.4 [5.9-11] | 8.4 [6.2-10] | 0.43 | 0.15 | 0.74 |
|  | 3 | 6.2 [5.1-11] | 8 [7-11] | 8.9 [5.3-11] | 9.1 [6.6-13] | 0.28 | 0.17 | 0.07 |
|  | 4 | 3.4 [3-5.4] | 8.8 [5.2-13] | 5.1 [2.9-5.7] | 6.7 [5.1-13] | 0.001 | 0.003* | 0.043* |
| ROI ventilation  (ml/min/g of lung tissue) | 1 | 6.4 [5.4 - 8.9] | 2.5 [1.6 - 3.1] | 9.0 [6.1 - 10.3] | 3.1 [2.2 - 4.8] | <0.001 | 0.008* | 0.001* |
|  | 2 | 12.5 [8.5 - 14.6] | 7.3 [5.7 - 8.2] | 10.3 [7.9 - 14.3] | 10.2 [5.1 - 11.5] | 0.005 | 0.013* | 0.10 |
|  | 3 | 8.6 [6.4 - 9.7] | 9.8 [6.7 - 12.2] | 8.1 [5.6 - 9.2] | 7.9 [7.1 - 12.2] | 0.007 | 0.20 | 0.007* |
|  | 4 | 3.9 [2.9 - 4.6] | 13.0 [10.1 - 14.9] | 3.1 [2.2 - 5.7] | 9.9 [6.6 - 12.7] | <0.001 | <0.001* | 0.002* |
| ROI perfusion  (ml/min/g of lung tissue) | 1 | 3.3 [2.5 - 4.6] | 3.3 [2.5 - 4.7] | 5.0 [1.7 - 6.7] | 3.8 [3.2 - 5.0] | 0.45 | 0.80 | 0.17 |
|  | 2 | 7.6 [5.6 - 8.2] | 8.1 [5.7 - 8.5] | 7.6 [5.0 - 8.0] | 9.1 [6.2 - 10.5] | 0.007 | 0.23 | 0.005* |
|  | 3 | 9.2 [6.6 - 13.4] | 7.7 [4.4 - 8.5] | 7.4 [5.4 - 9.1] | 6.8 [4.5 - 8.4] | 0.012 | 0.65 | 0.003* |
|  | 4 | 6.0 [3.2 - 9.5] | 6.0 [2.5 - 8.3] | 4.5 [2.7 - 6.1] | 4.8 [2.0 - 5.4] | 0.51 | 0.66 | 0.62 |
| Ventilation / Perfusion ratio | 1 | 2.1 [1.6 - 2.5] | 0.6 [0.5 - 1.1] | 1.8 [1.1 – 4.9] | 0.8 [0.5 - 1.3] | 0.014 | 0.15 | 0.029* |
|  | 2 | 1.6 [1.1 - 2.5] | 1.0 [0.9 - 1.1] | 1.7 [1.1 - 2.3] | 1.0 [0.8 - 1.3] | 0.001 | 0.003* | 0.025* |
|  | 3 | 0.9 [0.6 - 1.1] | 1.4 [1.2 - 1.7] | 1.1 [0.9 - 1.4] | 1.4 [1.3 - 1.7] | 0.001 | 0.003* | 0.044* |
|  | 4 | 0.6 [0.5 - 2.0] | 2.1 [1.8 - 4.8] | 0.8 [0.6 - 1.4] | 2.7 [1.6 - 4.3] | <0.001 | 0.001* | <0.001* |

Mixed-effects model analysis for global EIT data: ^†^ Positioning effect; ^‡^ Contrast estimate significance. *Significant difference between prone and supine using the same PEEP titration technique (p < 0.05). ROI: region of interest (1-vertebral; 4-dorsal); EIT: Electrical Impedance Tomography; PEEP: positive end-expiratory pressure.

**Supplement Table 4. Coefficients of variation across ROIs of key variables**

|  | ***PEEP set according to the***  ***ARDS Network low PEEP/FiO_2_ table (BP_ARDS_)*** | | ***PEEP set targeting the***  ***lowest transpulmonary driving pressure (BP_DPL_)*** | | ***P value*** | | |
| --- | --- | --- | --- | --- | --- | --- | --- |
|  | ***Supine*** | ***Prone*** | ***Supine*** | ***Prone*** | ***Position***^†^ | ***BP_ARDS_***^‡^ | ***BP_DPL_***^‡^ |
| Regional ventilation (CV %) | 42 [29 - 67] | 57 [45 - 63] | 49 [38 - 58] | 51 [37 - 62] | 0.75 | 0.64 | 0.97 |
| Regional perfusion (CV %) | 50 [30 - 68] | 36 [31 - 41] | 37 [26 - 42] | 41 [28 - 52] | 0.52 | 0.17 | 0.55 |
| Regional ventilation/perfusion ratio (CV %) | 62 [42 - 64] | 57 [46 - 82] | 53 [22 - 82] | 78 [22 - 81] | 0.63 | 0.72 | 0.75 |
| Regional strain (CV %) | 52 [49 - 56] | 21 [12 - 42] | 55 [52 - 63] | 19 [12 - 35] | <0.001 | <0.001* | <0.001* |

Mixed-effects model analysis for coefficients of variations across ROIs of selected variables: ^†^ Positioning effect; ^‡^ Contrast estimate significance. *Significant difference between prone and supine using the same PEEP titration technique (p < 0.05).
